# Prevalence of insertion sequence elements in plasmids relating to *mgrB* gene disruption causing colistin resistance in *Klebsiella pneumoniae*

**DOI:** 10.1002/mbo3.1262

**Published:** 2022-01-28

**Authors:** Stephen Mark Edward Fordham, Anna Mantzouratou, Elizabeth Sheridan

**Affiliations:** ^1^ Department of Life & Environmental Sciences Bournemouth University Poole UK; ^2^ Department of Medical Microbiology University Hospitals Dorset NHS Foundation Trust, Poole Hospital Poole UK

**Keywords:** antibiotic resistance, insertion sequence, *Klebsiella*, plasmid

## Abstract

Colistin is a last resort antibiotic for the treatment of carbapenemase producing *Klebsiella pneumoniae*. The disruption of the *mgrB* gene by insertion sequences (ISs) is a mechanism mediating colistin resistance. Plasmids encode mobilizable IS elements which integrate into the *mgrB* gene in *K. pneumoniae* causing gene inactivation and colistin resistance. The species prevalence of *mgrB*‐gene disrupting insertion elements ISL3 (ISKpn25), IS5 (ISKpn26), ISKpn14, and IS903B present on plasmids were assessed. IS containing plasmids were also scanned for antimicrobial resistance genes, including carbapenem resistant genes. Plasmids encoding ISs are abundant in *K. pneumoniae*. IS903B was found in 28 unique Inc groups, while ISKpn25 was largely carried by IncFIB(pQil) plasmids. ISKpn26 and ISKpn14 were most often found associated with IncFII(pHN7A8) plasmids. Of the 34 unique countries which contained any of the IS elements, ISKpn25 was identified from 26. ISKpn26, ISKpn14, and IS903B ISs were identified from 89.3%, 44.9%, and 23.9% plasmid samples from China. Plasmids carrying ISKpn25, ISKpn14, and ISKpn26 IS have a 4.6‐, 6.0‐, and 6.6‐fold higher carbapenemase gene count, respectively, relative to IS903B‐carrying plasmids. IS903B bearing plasmids have a 20‐, 5‐, and 5‐fold higher environmental source isolation count relative to ISKpn25, ISKpn14, and ISKpn26 bearing plasmids. ISKpn25 present on IncFIB(pQil) sourced from clinical settings is established across multiple countries, while ISKpn26, ISKpn14, and IS903B appear most often in China. Carbapenemase presence in tandem with IS elements may help promote an extensively drug resistant profile in *K. pneumoniae* limiting already narrow treatment options.

## Graphical abstract

*Klebsiella pneumoniae* is the dominant species carrying colistin‐resistance inducing insertion sequence elements ISKpn25, ISKpn26, ISKpn14, and IS903B on plasmids. ISKpn25, ISKpn26, ISKpn14 bearing plasmids have a high carriage of carbapenemase genes and are associated with clinical source isolation relative to IS903B. Clinically derived plasmids that harbor both carbapenemases and IS elements may engender an extensively drug‐resistant phenotype during colistin therapy in hospitals, limiting already narrow treatment options.

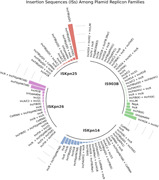

## 1 INTRODUCTION

Colistin serves as a last‐resort antibiotic choice for the treatment of bacterial infections caused by carbapenemase producing *Klebsiella pneumoniae* (CPKP) and other Gram‐negative isolates. Rising colistin use has seen a corresponding increase in colistin resistance, especially during therapy and is an emerging global threat. From 2011 to 2015, in Italy, the rate of colistin resistance in *Klebsiella pneumoniae* increased from 36% to 50% (Giani et al., 2015). Separately, in Thailand, colistin resistance has been reported at 76.1% from a sample of 280 *K. pneumoniae* clinical isolates collected from 2014 to 2017 (Eiamphungporn et al., 2018). Notably, colistin resistance due to disruption of the chromosomal *mgrB* gene in *K. pneumoniae* via the integration of insertion sequences (IS) has been widely reported from countries including: Lao PDR, Thailand, Nigeria, and France (Olaitan et al., 2014), Italy (Esposito et al., 2018), Greece (Giordano et al., 2018; Hamel et al., 2020; Zhu et al., 2019), Tunisia (Jaidane et al., 2018), Saudi Arabia (Zaman et al., 2018), Oman (Al‐Farsi et al., 2019), Israel (Lalaoui et al., 2019), India (Shankar et al., 2019), Taiwan (Yang et al., 2020), USA (Macesic et al., 2019), and Malaysia (Yap et al., 2020).

Colistin binds to the lipopolysaccharide (LPS) component of the outer membrane of Gram‐negative bacteria. The cationic diaminobutyric acid (Dab) residues of colistin bind to anionic phosphate groups in the LPS. Colistin then destabilizes both Mg^2+^ and Ca^2+^ divalent cations from the phosphate groups of LPS, disrupting the integrity of the membrane. Following membrane destabilization, colistin binds to the lipid A moiety of LPS causing the derangement of the outer membrane (Falagas et al., 2005). Colistin resistance in *K. pneumoniae* is mediated by the modification of the LPS through the addition of 4‐amino‐4‐deoxy‐l‐arabinose (l‐Ara4N) to the phosphate groups of the lipid A moiety. l‐Ara4N addition to LPS attenuates the affinity of colistin to LPS targets (Helander et al., 1996). The l‐Ara4N induced modification of LPS is controlled by the products of the *pmrHFIJKLM* operon, positively regulated by the two component PhoQ/PhoP and PmrAB systems. MgrB, a product of the *mgrB* gene is a small transmembrane regulatory protein synthesized following the activation of the PhoQ/PhoP signaling cascade. MgrB interacts with the PhoQ sensor kinase exerting negative feedback on the PhoQ/PhoP system (Lippa & Goulian, 2009). Insertional inactivation of *mgrB* prevents the downregulation of the PhoQ/PhoP systems and represents a mechanism facilitating de novo acquired colistin resistance (Cannatelli et al., 2013).

Resistance to colistin frequently arises via the disruption of the *mgrB* gene by ISs in *K. pneumoniae*. IS‐mediated *mgrB* gene disruption can represent a significant cause of colistin induced resistance in *K. pneumoniae*. Across two independent samples of 31 and 49 clinically isolated colistin resistant *K. pneumoniae* from Taiwan, 34.7% (*n* = 17/49), and 32.2% (*n* = 10/31) carried IS elements in the *mgrB* gene (Berglund et al., 2018; Yang et al., 2020), while 30% (*n* = 6/20) clinical colistin resistant *K. pneumoniae* isolates from Iran carried IS elements insertion, either IS5‐like or IS1‐like in *mgrB* (Haeili et al., 2017). More strikingly, from a sample of 11 clinical colistin resistant *K. pneumoniae* isolates from China, 9 carried IS elements in the *mgrB gene* (Yan et al., 2021), while 93.75% (*n* = 15/16) clinical isolates from Greece harbored ISKpn26‐like element disruption in *mgrB* (Zhu et al., 2019). ISs elements including ISKpn25, ISKpn26, IS903B, and ISKpn14 are common IS elements targeting genes involved in colistin resistance (Berglund et al., 2018; Di Tella et al., 2019; Haeili et al., 2017; Yan et al., 2021; Yang et al., 2020). Insertional inactivation of *mgrB* represents a prominent mechanism mediating the emergence of colistin resistance in *K. pnuemoniae*.

De novo colistin resistance may occur through the transposition of ISs from plasmids into chromosomal colistin‐associated genes. Across a sample of 29 and 19 colistin resistant *K. pneumoniae* isolates from Italy and Greece, 2 clonally related clusters; 2 Italian ST512 isolates, and 8 Greek ST258 *K. pneumoniae* isolates harbored the complete copy of ISKpn25 inserted at nucleotide position 133 of the *mgrB* gene (Giordano et al., 2018). The same ISKpn25 was located on pKpQIL‐like plasmids from these samples, indeed the ISKpn25 on pKpQIL plasmids and the ISKpn25 inserted into the *mgrB* gene share a 100% match between the 8154 nucleotides. Furthermore, in two ST258 and two ST512 *K. pneumoniae* isolates from Greece and Italy, the IS5‐element derived from pKpQIL‐like plasmids, was found inserted into nucleotide position 75 of the *mgrB* gene (Giordano et al., 2018). Nucleotide position 75 represents a hotspot for IS5 element insertion among clonally unrelated *K. pneumoniae* isolates (Cannatelli et al., 2013; Poirel et al., 2014). Mobilization of IS5 elements from plasmids into the *mgrB* gene of colistin resistant *K. pneumoniae* has been speculated owing to the similarity between the IS5 element inserted into *mgrB* and the IS5 element present on *K. pneumoniae* carbapenemase (KPC)‐encoding and other Gram‐negative bacterial plasmids (Azam et al., 2021; Cannatelli et al., 2013; Hala et al., 2019), and endogenous presence of IS5‐like elements in the genome of colistin resistant *K. pneumoniae* (Choi & Ko, 2020).

Further evidence for the involvement of plasmids as donors for IS elements is provided by a recent cloning assay. IS elements including ISKpn26, ISKpn14, and IS903B cloned into a plasmid vector and transformed into a colistin susceptible *K. pnuemoniae* isolate increased the frequency of colistin resistance *K. pneumoniae* isolates. Notably, for the plasmid vector carrying IS903B, colistin induced stress was responsible for IS mobilization (Yang et al., 2020). Furthermore, a *Caenorhabditis elegans* killing assay model revealed nematodes fed with *K. pneumoniae* isolates harboring plasmids carrying ISKpn26 were associated with a significantly reduced lifespan and higher death risk during colistin treatment relative to nematodes fed with a non‐IS plasmid carrying *Escherichia coli* and *K. pneumoniae* isolates, thus confirming the role of plasmids as donors for IS elements mediating colistin resistance (Yang et al., 2020).

Beyond gene insertion, IS elements can integrate into the promoter regions of chromosomal colistin‐resistance associated genes. The IS1R IS element sourced from a plasmid has been shown to integrate into the promoter region of *mgrB* mediating the emergence of a de novo colistin resistant phenotype (Antonelli et al., 2017), indeed IS1‐like element insertion has been independently reported repeatedly in the promoter region of *mgrB* (Berglund et al., 2018; Haeili et al., 2017). For IS1382‐like and IS1‐like elements disrupting *mgrB* in *K. pneumoniae*, BLASTn searches revealed their presence only in respective isolates with disruptions in *mgrB*, notably absent from any other chromosomal location, thereby indicating a likely plasmid source as opposed to transposition from a chromosomal source (Jaidane et al., 2018).

Colistin resistance may arise through the transposition of ISs from source IS containing plasmids preferentially targeting specific *mgrB* regions for recombination. The dissemination of IS elements that transpose into the same position of the *mgrB* gene may represent a mechanism mediating the observed colistin resistance in both clonally related and unrelated *K. pneumoniae* isolates. IS elements are frequently reported in *K. pneumoniae* colistin‐associated chromosomal resistant genes. Examples of ISKpn25, ISKpn26, ISKpn14, and IS903B inserted into the *mgrB* gene of *K. pneumoniae* are shown in Table A1. To ascertain IS element prevalence in *K. pneumoniae*, IS elements which have been shown to disrupt the *mgrB* gene; namely ISKpn25, ISKpn26, ISKpn14, and IS903B were investigated. Species IS prevalence among 1000 BLASTn hits was assessed. Separately, to support pathogen surveillance, plasmid incompatibility typing (Inc) for the top 120 BLASTn plasmid hits was performed to determine the respective plasmid incompatibility group associated with each IS element. In addition, metadata for the same plasmid samples were gathered to determine dual carbapenemase gene prevalence, plasmid size, species, country, and isolation source to elucidate the emerging clinical threat posed by plasmid bearing IS elements.

## 2 METHOD

Four IS reference elements were derived from the online IS finder tool (https://www-is.biotoul.fr). The IS elements include: IS1 (ISKpn14), ISL3 (ISKpn25), IS5 (ISKpn26), and IS903B ISs (Table 1a). Each IS element was blasted using the NCBI nucleotide blast online tool, BLASTn. To assess IS proportional assignment between bacterial species, the max target sequences parameter was increased from the default of 100–1000 for each IS element. The description table for each BLASTn hit was downloaded and filtered for plasmids to ascertain plasmid samples encoding IS elements. For ISKpn25 which returned many low identity and coverage hits, plasmid hits above a minimum threshold; percentage identity ≥95% was used. This threshold was chosen to differentiate ISKpn25 from ISKpn26 which shares 91% nucleotide identity (https://www-is.biotoul.fr). The relative proportion of IS elements among *K. pneumoniae* samples versus other bacterial species was calculated and plotted.

**Table 1a:** Reference IS elements soured from IS finder with their associated length, open reading frame, open reading frame function, and accession numbers

Reference IS element	Length (bp)	Accession number	Open reading frame(s)	Open reading frame function
IS1 (ISKpn14)	768	CP000649	91 (56–331)	1
			174 (229–753)	1
			274 (56–753)	1
ISL3 (ISKpn25)	8154	NC_009650	684 (107–2161)	1
			438 (2158–3474)	2
			1085 (3475–6732)	3
			422 (6737–8005)	4
IS5 (ISKpn26)	1196	NC_016845	326 (69–1049)	1
IS903B	1057	X02527	307 (78–1001)	3
			70 (672–460)	1

*Note*: 1, transposase; 2, putative restriction endonuclease S subunit; 3, hypothetical protein; 4, putative type I restriction‐modification system DNA methylase.

Abbreviation: IS, insertion sequence.

**Figure 1:**
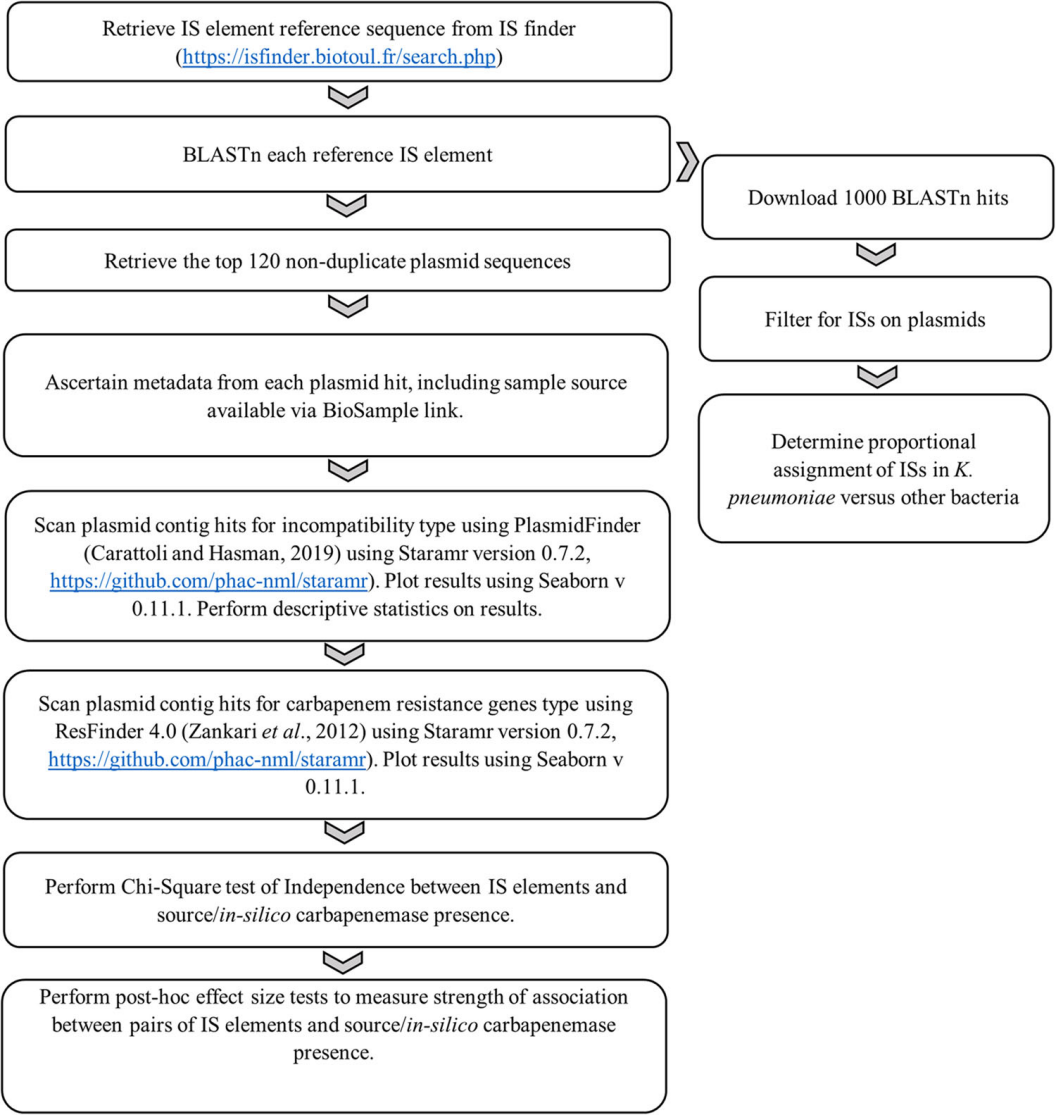
Bioinformatics method workflow. National Microbiology Laboratory, Staramr (n.d.)

For each IS element, the top 120 nonduplicate circularized plasmid hits harboring IS elements were downloaded, and the Fasta contigs incompatibility (Inc) typed with PlasmidFinder using a minimum identity and length of 98% (Carattoli & Hasman, 2019). Each plasmid contig carrying IS elements was also scanned for carbapenemase genes with a predicted in silico resistance phenotype to the carbapenem, meropenem and other resistant genes using ResFinder 4.0 with a minimum identity and length of 98% (Zankari et al., 2012). Metadata pertaining to each sample, including accession number, species and isolation source, country of origin, and plasmid size was recorded by accessing available Biosample information from the BLASTn hits from NCBI. Results were tabulated and analyzed using Python and are available in supplementary material at https://doi.org/10.5281/zenodo.5812148. Figures were produced using both the Matplotlib and Seaborn libraries in Python. For statistical analyses between the IS elements and the presence of carbapenemase genes/sample source, the *χ* ^2^ test of independence was used with an *α* value of 0.05 as the significance level. To correct against multiple comparisons between each IS element, *p*‐values were corrected using the false discovery rate (FDR) method. To measure the strength of association between carbapenemase gene prevalence/clinical source isolation against different IS elements, Cramer's *V* was calculated for each pair of IS elements. Statistical analyses were conducted using IBM SPSS Statistics v28.0.0. The criterion outlined in Table 1b was used for Cramer's *V* effect size characterization (IBM, 2021). The method workflow is shown in Figure 1.

**Table 1b:** Cramer's *V* is an effect size measure used for the *χ* ^2^ test of independence

Effect size (ES)	Interpretation
ES ≤ 0.2	Fields are weakly associated
0.2 < ES ≤ 0.6	Fields are moderated associated
ES > 0.6	Fields are strongly associated

*Note*: Cramer's *V* measure the strength of association between two categorical fields.

For clinical sample classification, biosample data labeled as: blood, urine, human hospital, rectum, feces, sputum, necrotic tissue, throat, wound swab, clinical sample, hospital environment, ascites, bile, tissue sample, wound, abdominal pus, secreta, stool, lymphocele, intestine fluid, groin, abscess, bronchoalveolar lavage, and ulcers were classified as clinically derived samples, while samples labeled as environment, pig, horse, intestines (animal), sample milk, sewage, *Manis javanica*, waste treatment plant, food, equine body fluid, and dog were classified as environmentally derived samples in our analysis.

Biosamples termed wastewater, wastewater influent sample, and wastewater effluent samples are not categorized as either clinical samples or environmental samples. A total of 21 samples fit into this third undetermined category. These samples do not feature in the frequency data pool used in both the *χ* ^2^ test of Independence between IS elements and sample source, and the subsequent post hoc Cramer's *V* association effect size tests. The sample source for each plasmid can be found in supplementary material at https://doi.org/10.5281/zenodo.5812148.

## 3 RESULTS

### 3.1 IS element pervasiveness in *K. pneumoniae*

**Figure 2:**
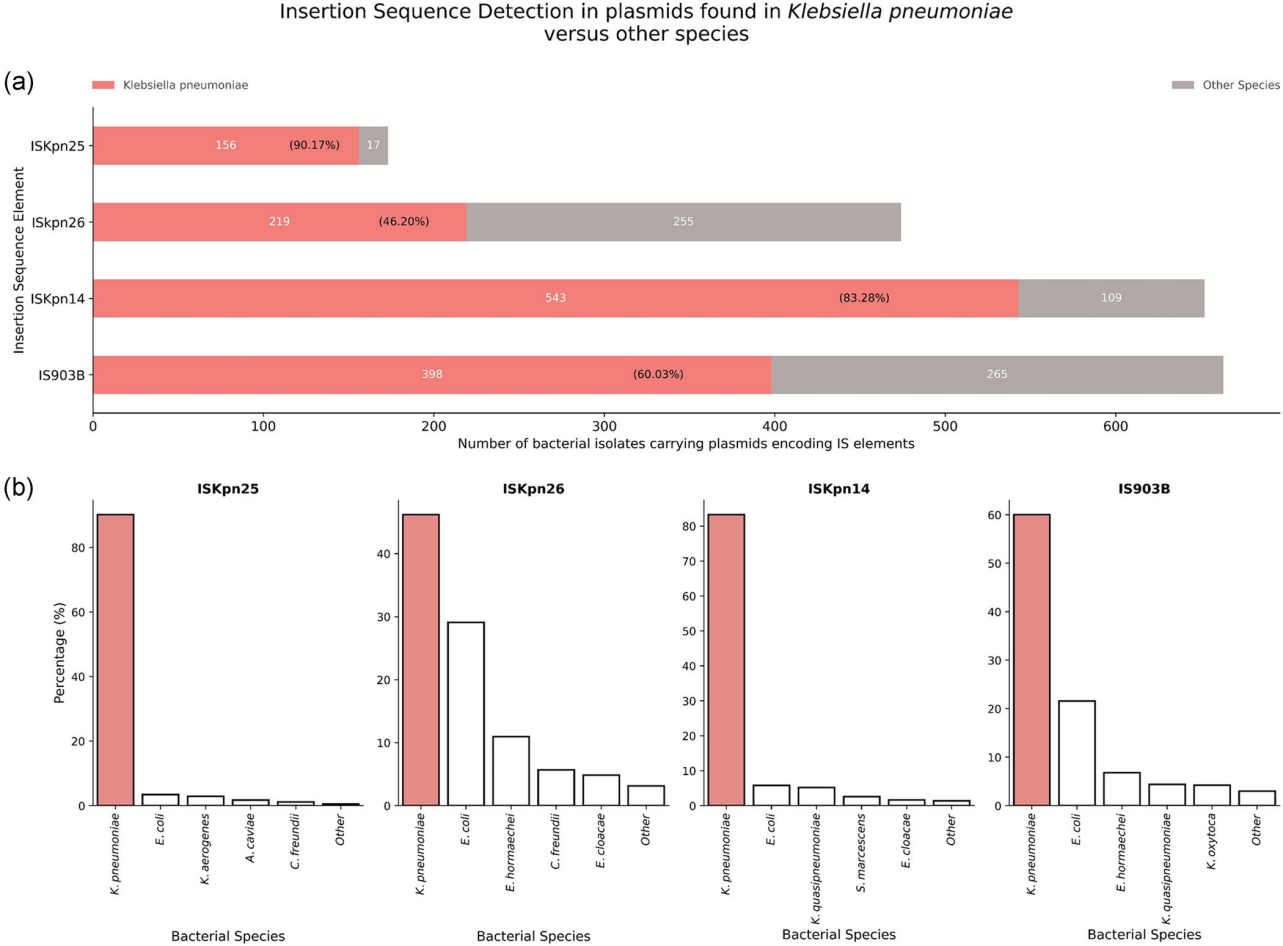
Proportional assignment of insertion sequence (IS) elements ISKpn25, ISKpn26, ISKpn14, and IS903B among *Klebsiella pneumoniae* and other bacterial species. (a) The max target sequences parameter on the NCBI BLASTn tool (https://blast.ncbi.nlm.nih.gov) was adjusted from the default of 100 to 1000, to gain insight into IS proportional assignment among species. For each IS element, *K. pneumoniae* plasmid hits are shown as red horizontal bars with bacterial counts indicated in white, percentages in parentheses in black. (b) *K. pneumoniae* bars shaded in red depict species percentage; the closest four species are given a separate white bar, while the reminding species are grouped into a single white bar labeled other

*K. pneumoniae* represents the dominant species harboring IS elements; ISKpn25, ISKpn26, ISKpn14, and IS903B on plasmids. Across the sample of plasmids encoding ISKpn25 (*n* = 173, mean query coverage: 99.85%, standard deviation: 0.92%, mean identity: 99.87%, standard deviation: 0.73% against reference NC_009650), *K. pneumoniae* constituted 90.17% (*n* = 156) of all samples carrying ISKpn25 (Figure 2a, ISKpn14). In addition, *K. pneumoniae* carried the IS element ISKpn25 at a 31.2‐fold higher rate than the second most abundant bacterium encoding ISKpn25 on plasmids, namely *E. coli* (Figure 2b, ISKpn25).

*K. pneumoniae* also represents the most common species carrying the IS element ISKpn26 on plasmids. Across the sample of plasmids encoding ISKpn26 (*n* = 474, mean query coverage: 100%, mean identity: 99.78%, standard deviation: 0.13% against reference NC_016845), *K. pneumoniae* comprised 46.20% of plasmid samples carrying ISKpn26, a rate 4.2‐fold higher than *E. coli* (Figure 2a,b, ISKpn26).

Across a sample of 652 plasmid samples encoding ISKpn14 (mean query coverage: 100%, mean identity: 99.98%, standard deviation: 0.11% against reference CP000649), *K. pneumoniae* was the principal bacterium carrying ISKpn14 on plasmid samples, responsible for 82.3% (*n* = 543) of ISKpn14 plasmid samples (Figure 2a, ISKpn14). *K. pneumoniae* carried ISKpn14 at a rate 15.9‐fold higher relative to *E. coli*, the second most abundant ISKpn14‐carrying species (Figure 2b, ISKpn26).

Finally, in line with observations for the other three IS elements, *K. pneumoniae* was the dominant species encoding IS903B‐like IS elements on plasmids. IS903B‐like ISs were found on 60.03% (*n* = 398) of IS903B‐plasmid containing samples (mean query coverage: 99.99%, standard deviation: 0.03%, mean identity: 98.72%, standard deviation: 0.21% against reference X02527). *K. pneumoniae* harbored IS903B at an 8.84‐fold higher rate relative to *E. coli*, the second most abundant species encoding IS903B (Figure 2b, IS903B).

### 3.2 IS element stratification among plasmid incompatibility groups

**Figure 3:**
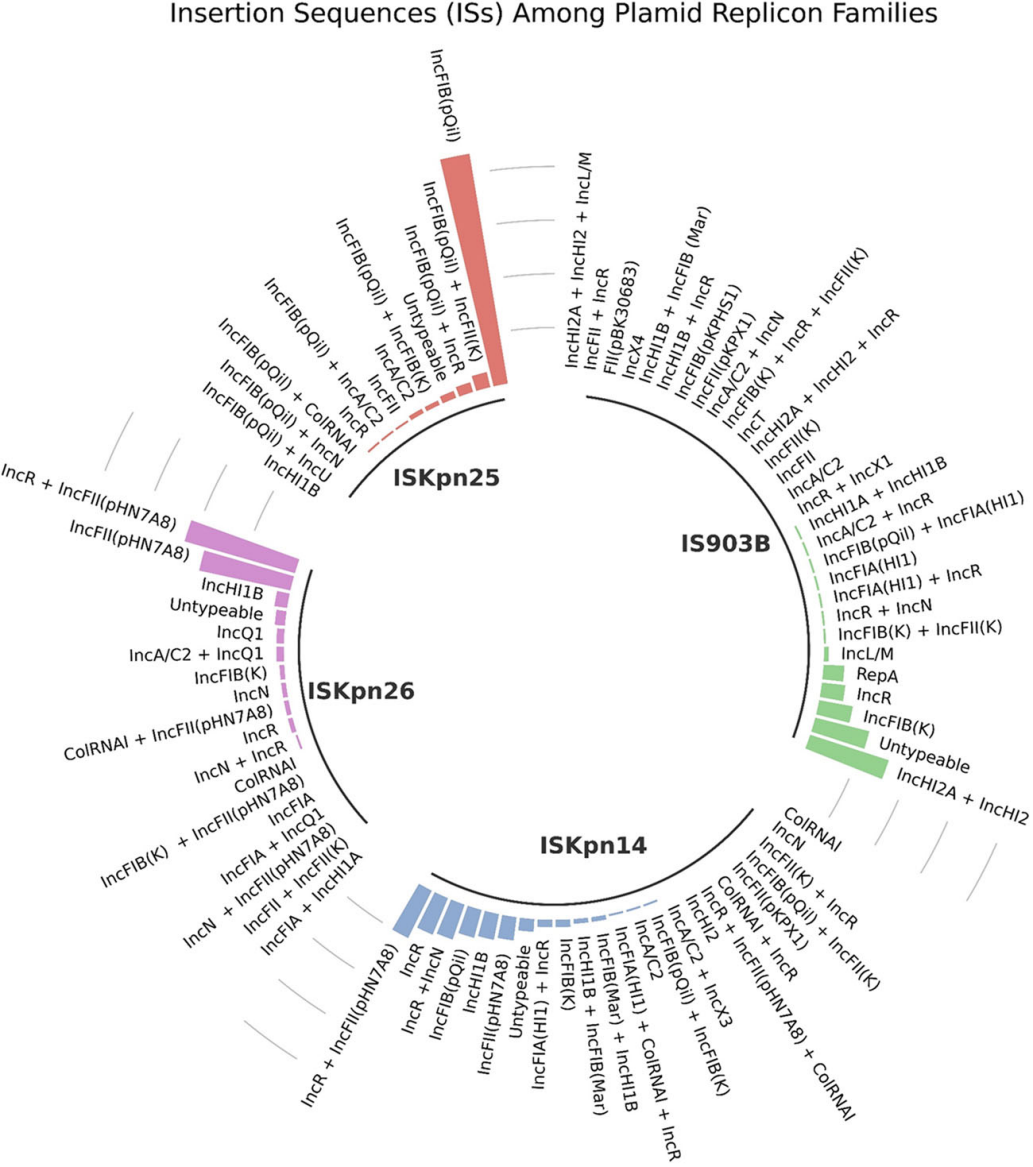
Insertion sequences (ISs) stratification among plasmid replicon families. IS elements identified in plasmids belonging to various plasmid incompatibility (Inc) groups. The circular bar plot schematic represents the results of the top 120 IS BLASTn hits for each insertion element present in plasmids (accession numbers for IS elements listed in Section 2, Table 1a). Relative to ISKpn25 colored red, identified in 12 unique Inc groups, ISKpn26 colored purple, is present in 17 unique Inc groups, 1.42‐fold higher), ISKpn14 colored blue, is present in 22 unique Inc groups, 1.83‐fold higher, while IS903B, colored green, is the most diverse plasmid host‐range IS element, present in 28 unique Inc groups, with a 2.33‐fold higher plasmid replicon count. The dominant Inc groups per IS are IncFIB(pQil) for ISKpn25 IS elements, the fusion plasmid IncHI2A + IncHI2 for IS903B IS elements and the fusion plasmid IncR + IncFII(pHN7A8) for ISKpn26 and ISKpn14 IS elements, respectively. Plasmids which were classified as untypeable were not regarded as a unique Inc group

The four IS elements follow a different stratification pattern among plasmid incompatibility groups (Inc). The IS element, IS903B is the most diverse plasmid host range element, present in 28 unique Inc groups or Inc groups representing fusion plasmids. IS903B was present in 1.42‐, 1.83‐, and 2.33‐fold higher unique Inc groups, relative to ISKpn14, ISKpn25, and ISKpn26, respectively (Figure 3). Despite the broad host range of IS903B among various unique plasmid Inc groups, 16/28 represented a single instance of the IS element associated with a unique plasmid Inc group. Indeed, for the other IS elements investigated, a single Inc group was found in 9, 7, and 4 instances for the IS elements ISKpn14, ISKpn26, and ISKpn25, respectively. To determine the dominant Inc groups associated with the IS elements, the data set was filtered for ≥5 occurrences of the same Inc group. Here, 3, 3, 4, and 6 unique Inc groups were identified in plasmids harboring ISKpn25, ISKpn26, IS903B, and ISKpn14 elements, respectively. Notably, the IncFIB(pQil) Inc group was present in 88/120 samples harboring ISKpn25. Furthermore, the same IncFIB(pQil) Inc group in addition to either IncR or IncFII(K) was found in an additional five and seven plasmids encoding the insertion element ISKpn25. From the top 120 BLASTn hits, the ISKpn25 element is disproportionately identified in the plasmid belonging to the IncFIB(pQil) replicon family. Likewise, plasmids harboring the IS elements, ISKpn26 and ISKpn14 follow a pronounced partitioning among Inc groups. The ISKpn26 elements were associated with IncFII(pHN7A8) encoding plasmids. Here, IncFII(pHN7A8) was found in *n* = 36/120 plasmids while *n* = 44/120 plasmids carried both IncFII(pHN7A8) and the IncR group. ISKpn14 was associated with the IncHI2A and IncHI2 fusion groups. In contrast, the IS element IS903B is associated with various plasmid Inc groups including IncHI2A + IncHI2, IncFIB(K), IncR and RepA, as shown in Figure 3. Taken together, IS elements with demonstrable ability to disrupt chromosomal colistin‐associated genes are present in a large number, namely 61 unique Inc groups from a sample size of 480 BLASTed IS elements. Despite this, specific Inc groups are disproportionately associated with particular IS elements. Typing plasmids may help to yield the relationship between IS elements and their partner plasmid families.

**Figure 4:**
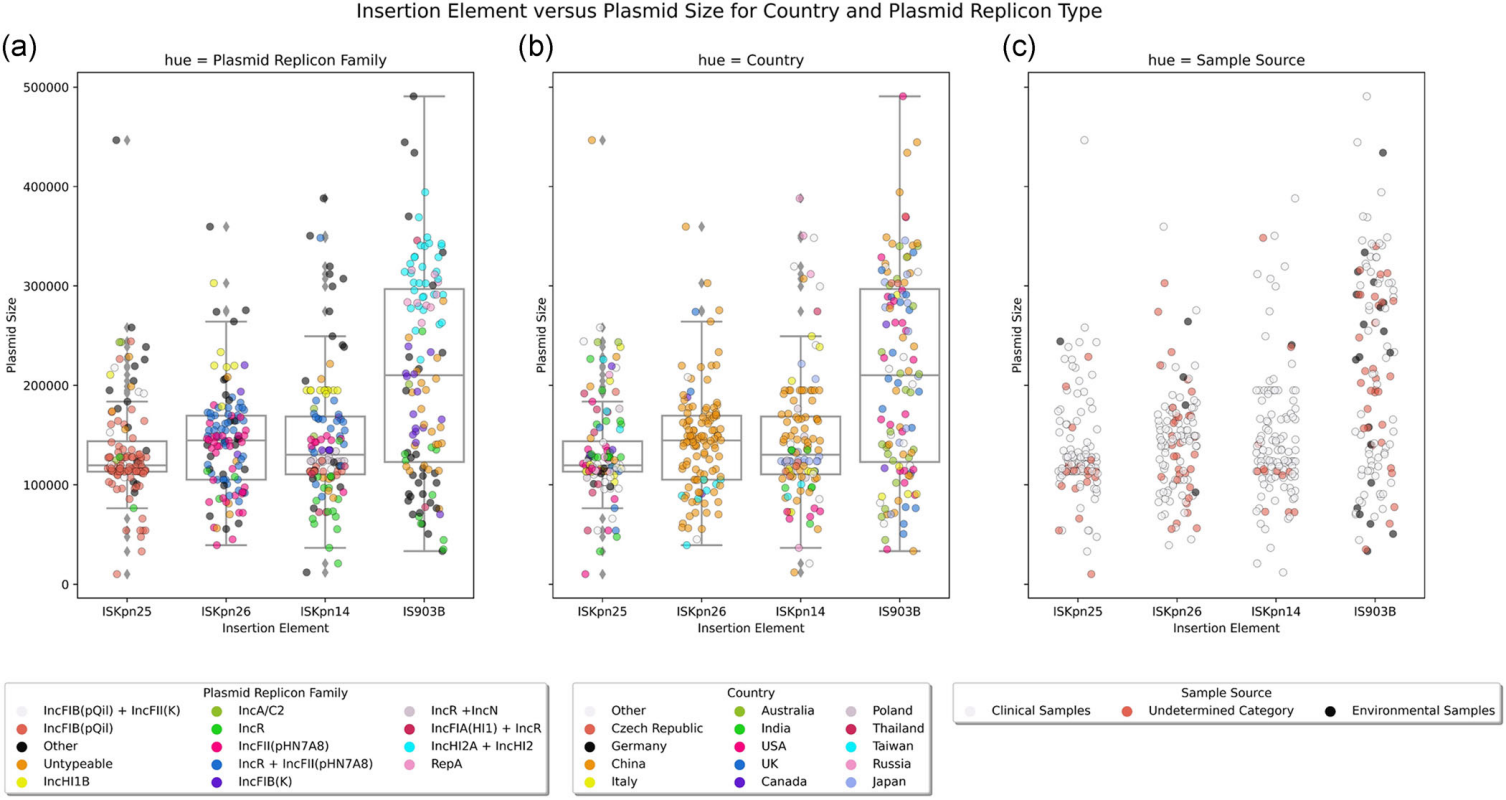
Insertion element stratification for ISKpn25, ISKpn26, ISKpn14, and IS903B. (a) Plasmid replicon family versus plasmid size for the four IS elements. (b) Country stratification versus plasmid size. (c) Source sample stratification. Each IS element included 120 nonduplicate plasmid samples. Plot produced using the Seaborn library in Python. IS, insertion sequence

The four IS elements are associated with diverse plasmids with a varied size ranging from 10,159 bp through to 490,750 bp (Figure 4). Notably, of the 34 unique countries which contained any of the IS elements, ISKpn25 was identified from 26. India, the USA, and Italy were the country of origin for 16.6%, 13.3%, and 9.2% of ISKpn25 insertion element plasmid samples. In contrast, typed ISKpn26 elements are associated with IncFII(pHN7A8) Inc groups, and are largely restricted to China, 89.3% (*n* = 107/120), with a further seven samples identified from Taiwan. Similar to ISKpn26 encoding elements, the largest proportion of typed samples encoding ISKpn14, 44.9%, were isolated from China. Finally, China represents the single largest country harboring IS903B samples, with a 23.9% proportional assignment, however IS903B is also found at similar levels in the United States (18.8%), Australia (18.8%), and the United Kingdom (14.5%), suggesting IS903B is established internationally.

Across the four IS elements, 74.16% (*n* = 356/480) typed plasmid samples were sourced from clinical samples. China is the largest country harboring ISKpn26 (*n* = 73/83, 87.9%) and ISKpn14 (*n* = 53/107, 49.5%) clinical samples, while China is the second largest contributor for IS903B derived clinical samples (*n* = 18/68, 26.5%). In contrast, the clinically sourced ISKpn25 samples are derived from 24 countries, although India contributes 20.6% of these samples. Taken together, IS elements present in plasmids are endemic in China, established in other countries and both the environment and clinical settings may represent sources for IS elements. Notably, the environment may serve as a potential origin for plasmid encoded IS903B samples whereby 19.05% (*n* = 20/105) of plasmids encoding this IS element are derived from environmental sources, and a further 16.2% (*n* = 17/105) did not disclose an isolation source. Collectively, these relationships are summarized in Figure 4c.

### 3.3 Plasmids encoding both IS elements and carbapenemase genes

**Figure 5:**
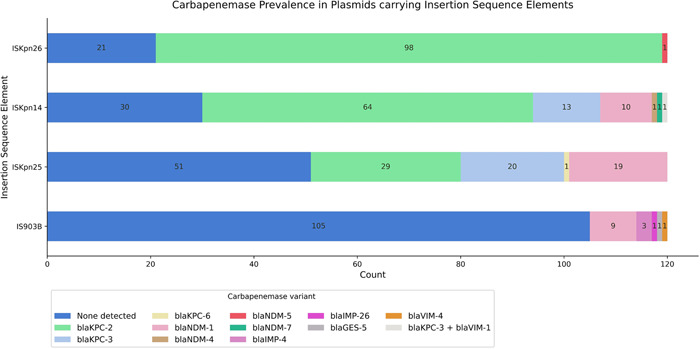
Carbapenemase prevalence in plasmids carrying IS elements. Plasmids encoding IS elements were also investigated for the presence of carbapenemase genes by scanning plasmid contigs against the ResFinder 4.0 database. Carbapenemase gene detection was based on the predicted in silico resistance phenotype to the carbapenem, meropenem. Figure produced using the Matplotlib library in Python. IS, insertion sequence

Plasmids carrying IS elements were additionally investigated for the presence of carbapenemase genes. A marked stratification pattern exists for the various IS elements and carbapenemase genes. For plasmids harboring either ISKpn26, ISKpn14, or ISKpn25, 82.5%, 75%, and 57.5% also carried carbapenemase genes with a predicted in silico resistance phenotype against the carbapenem, meropenem. In contrast, only 12.5% of plasmids carrying the IS element IS903B also carried carbapenemase genes (Figure 5). The *χ* ^2^ test of independence revealed a significant difference between carbapenemase gene distribution between the four IS elements, *χ* ^2^(3, *N* = 480) = 144.55, *p* ≤ 0.001. Post hoc analysis revealed a significant difference between IS903B, and the three other IS elements. Relative to IS903B, carbapenemase gene distribution was significantly different relative to ISKpn25, ISKpn26, and ISKpn14: IS903B versus ISKpn25 (*χ* ^2^, *p* < 0.001), IS903B versus ISKpn14 (*χ* ^2^, *p* < 0.001), and IS903B versus ISKpn26 (*χ* ^2^, *p* < 0.001) Results are summarized in Table 2a, 2b, 2c. A statistically significant difference between the distribution of sample source for IS elements was also observed, *χ* ^2^(3, *N* = 382) = 46.97, *p* ≤ 0.001. A significant difference between the distribution of sample source for IS903B against ISKpn14 (*χ* ^2^, *p* < 0.001), ISKpn26 (*χ* ^2^, *p* < 0.001), and ISKpn25 (*χ* ^2^, *p* < 0.001) was observed. Results are summarized in Table 2d.

**Table 2a:** The association between in silico carbapenemase gene presence and IS elements

		IS903B		
IS element	Cramer's *V* (*φ*_c_)	Carbapenemase fold difference	*p*‐value	Pearson's *χ* ^2^
ISKpn25	0.472	x4.6 higher (ISKpn25)	<0.001	*χ* ^2^ (1, *N* = 240) = 53.407
ISKpn14	0.630	x6.0 higher (ISKpn14)	<0.001	*χ* ^2^ (1, *N* = 240) = 95.238
ISKpn26	0.701	x6.6 higher (ISKpn26)	<0.01	*χ* ^2^ (1, *N* = 240) = 117.895

*Note*: IS903B versus ISKpn25, ISKpn14, and IS5 (ISKpn26), significant results (*p* < 0.05) presented only.

**Table 2b:** The association between in silico carbapenemase gene presence and IS elements

		ISKpn25		
IS element	Cramer's *V* (*φ*_c_)	Carbapenemase fold difference	*p*‐value	Pearson's *χ* ^2^
ISKpn26	0.273	x1.43 higher (ISKpn26)	<0.001	*χ* ^2^ (1, *N* = 240) = 17.857

*Note*: ISKpn25 versus ISKpn26.

**Table 2c:** The association between in silico carbapenemase gene presence and IS elements

		ISKpn25		
IS element	Cramer's *V* (*φ*_c_)	Carbapenemase fold difference	*p*‐value	Pearson's *χ* ^2^
ISKpn14	0.185	x1.304 higher (ISKpn14)	<0.001	*χ* ^2^ (1, *N* = 240) = 8.218

*Note*: ISKpn14 versus ISKpn25.

**Table 2d:** The association between clinical source and IS elements

		IS903B			
IS element	Cramer's *V* (*φ*_c_)	Environment fold difference	Clinical fold difference	*p*‐value	Pearson's *χ* ^2^
ISKpn25	0.343	x20 higher (IS903B)	x1.44 higher (ISKpn25)	<0.001	*χ* ^2^ (1, *N* = 187) = 22.041
ISKpn14	0.351	x20 higher (IS903B)	x1.57 higher (ISKpn25)	<0.001	*χ* ^2^ (1, *N* = 196) = 24.992
IS5 (ISKpn26)	0.264	x5 higher (IS903B)	x1.22 higher (ISKpn25)	<0.01	*χ* ^2^ (1, *N* = 175) = 12.151

*Note*: IS903 versus ISKpn25, ISKpn14, and ISKpn26, significant results (*p* < 0.05) presented only.

Cramer's *V* (*φ*_c_) reveals a strong or moderate association (0.2 < ES ≤ 0.6/ES > 0.6) between IS elements (IS903b‐paired IS elements) and in silico carbapenemase gene presence (Table 1a). Plasmids carrying ISKpn25, ISKpn14, and ISKpn26 IS elements have a 4.6‐, 6.0‐, and 6‐fold higher carbapenemase count relative to plasmids carrying the IS element IS903B. Results are summarized in Table 2a. In addition, Cramer's *V* determined a moderate effect size (*φ*_c_ = 0.273; 0.2 < ES ≤ 0.6) between the paired IS elements ISKpn26, ISKpn25, and in silico carbapenemase gene presence, where ISKpn26 has a 1.43‐fold higher carbapenemase count relative to ISKpn25 (Table 2b). The weak effect size was recorded between the paired IS elements ISKpn14, ISKpn25, and in silico carbapenemase gene presence; *φ*_c_ = 0.165; ES ≤ 0.2, where ISKpn14 has a 1.3‐fold higher carbapenemase presence (Table 2c).

Cramer's *V* also revealed a moderate association between IS903b‐paired IS elements and source isolation. Relative to ISKpn26, ISKpn25, and ISKpn14, IS903B has a 5‐, 20‐, and 20‐fold higher environmental source isolation. Despite IS903B being more commonly detected from environmental sources, plasmids encoding IS903B are still found at a rate of 3.40 times higher from clinical sources. ISKpn26, ISKpn25, and ISKpn14 are however sourced from clinical sources, 20.75‐, 98‐, and 107‐fold higher relative to IS903B. These relationships are summarized in Table 2d.

**Figure A1:**
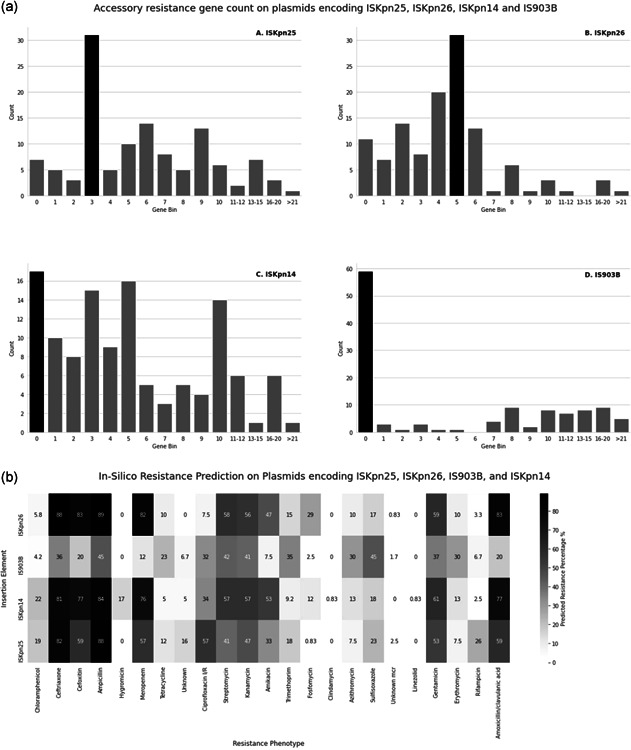
(a) Accessory gene count and average predicted resistance phenotype for plasmids encoding ISKpn25, ISKpn26, ISKpn14, and IS903B. Plasmid contigs were interrogated for resistance genes and predicted resistance phenotype using ResFinder 4.0 (using Staramr version 0.7.2, https://github.com/phac-nml/staramr). (a) The total number of resistance genes was counted and divided into 15 gene count bins to produce a histogram distribution plot. (b) The average predicted resistance percentage for the 23 detected antibiotics across the four insertion sequence elements: ISKpn25, ISKpn26, ISKpn14, and IS903B. The 23 antibiotics include chloramphenicol, ceftriaxone, cefoxitin, ampicillin, hygromycin, meropenem, tetracycline, unknown, ciprofloxacin I/R, streptomycin, kanamycin, amikacin, trimethoprim, fosfomycin, clindamycin, azithromycin, sulfisoxazole, unknown mcr, linezolid, gentamicin, erythromycin, rifampicin, and amoxicillin/clavulanic acid. Group unknown corresponds to a predicted phenotype of “unknown[catb4_af322577].” Figure produced using Seaborn v 0.11.1

**Figure A2:**
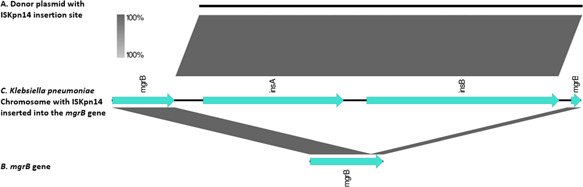
The number of resistant genes on plasmids stratified by insertion sequence element. (a) Boxplot of the number of resistant genes encoded on plasmids harboring either ISKpn25, ISKpn26, ISKpn14, or IS903B. IS903 has a larger standard deviation for the number of resistant genes mean = 5.69, standard deviation = 6.94, relative to ISKpn25 (mean = 6.06, stdv = 4.25), ISKpn26 (mean = 4.57, stdv = 3.47), and ISKpn14 (mean = 5.46, stdv = 4.66), respectively, reflecting plasmids with no resistant genes, and those with ≥10 resistance genes. Figure produced using Seaborn v 0.11.1

Moreover, the distribution of all resistance genes among the four IS elements was analyzed using the *χ* ^2^ test of independence. For all four IS elements, the number of plasmids encoding either 0, ≥2, ≥5, and ≥10 resistance genes was counted. A significant difference between the distribution of the total number of resistance genes among plasmids between the four IS elements was determined, *χ* ^2^(6, *N* = 480) = 108.125, *p* ≤ 0.001. A histogram and boxplot of the total number of resistance genes for each IS element is shown in Figures A1 and A2. Post hoc analyses compared each IS element against one another separately. Significant values were corrected using the FDR error correction method. Here, IS903B, had a significantly different distribution for the total number of resistant gene count, relative to ISKpn14 (*p* ≤ 0.001), ISKpn25 (*p* ≤ 0.001), and ISKpn26 (*p* ≤ 0.001), respectively. Notably, ISKpn14 and ISKpn26 also had a significantly different distribution between the total number of resistant genes detected (*p* ≤ 0.001). For IS903B, 49.16% (*n* = 59/120) of plasmids harbored no resistant genes, compared against 14.16% (*n* = 17/120), 11.0% (*n* = 11/120), and 5.83% (*n* = 7/120) for ISKpn14, ISkpn26, and ISkpn25, respectively. Furthermore, across the four IS elements, resistance to a total of 23 antibiotics was detected. Notably, across the 23 antibiotics, IS903B had the lowest predicted in silico resistance for 56.52% (*n* = 13/23) of the antibiotics (Figure A1b).

## 4 DISCUSSION

**Figure A3:**
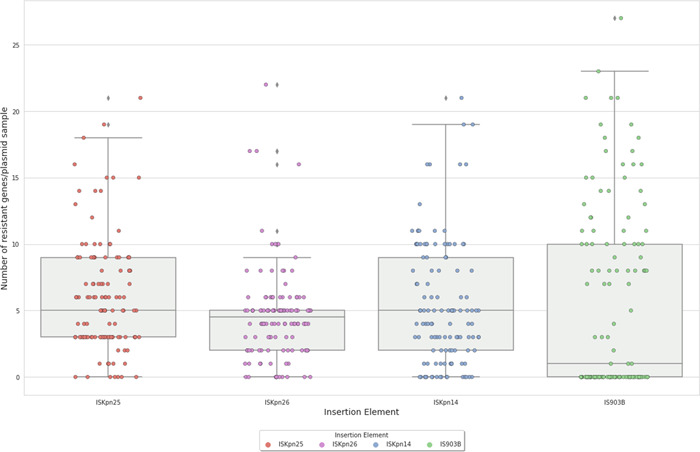
Proposed mechanism for the emergence of colistin resistance in *Klebsiella pneumoniae* via insertion sequence (IS)‐mediated *mgrB* gene disruption. Donor plasmids encoding ISs may represent a source of IS elements mediating colistin resistance. (a) The donor plasmid encodes the ISKpn14 IS element, ISKpn14 tr refers to the transposase encoded by ISKpn14. (b) ISKpn14 inserts into the *mgrB* gene, causing gene disruption. (c) Representative *K. pneumoniae* chromosomal sequence with ISKpn14 inserted at position +123 of the *mgrB* gene. The reference ISKpn14 element (accession: CP000649) on plasmids shares 100% nucleotide identity and coverage with the ISKpn14 element encoded in the chromosome of a *K. pneumoniae* isolate (accession: KJ129604) with a disrupted *mgrB* gene. Reference ISKpn14 with 100% nucleotide identity and coverage is found on a large number of divergent plasmids, such as CP054781, AP024793, MW36397, LC521840, FR997880 (Supporting Information at https://doi.org/10.5281/zenodo.5812148). These plasmids may serve as donors for ISKpn14 mediating colistin resistance via *mgrB* gene disruption. Figure produced using EasyFig version 2.2.2 (Sullivan et al., 2011)

*K. pneumoniae* represents a key reservoir species harboring plasmids encoding IS elements which could disrupt the *mgrB* gene leading to colistin resistance. The high prevalence of these four IS elements from plasmids derived from *K. pneumoniae* provides indirect evidence for their role in generating colistin resistance. The ISKpn14 reference element inserted into the chromosomal *mgrB* gene in a *K. pneumoniae* isolate (accession: KJ129604.1) has a 100% nucleotide identity and coverage match with the ISKpn14 element found on a wide range of plasmids, indicating the role of plasmids as possible donors for ISKpn14 IS elements. This relationship is depicted in Figure A3. Moreover, ISKpn25 (ISL3) encoding pKpQil‐like plasmids have been proposed as the donor for ISKpn25 elements. A 100% identity match between the ISKpn25 found on plasmids and the ISKpn25 disrupting the chromosomal *mgrB* gene has been reported across two *K. pneumoniae* STs: ST258, and ST512 in strains carrying both pKpQil‐like plasmids and a disrupted chromosomal *mgrB* gene across independent investigation of colistin resistant *K. pneumoniae* (Cienfuegos‐Gallet et al., 2017; Giordano et al., 2018). ISKpn26 and IS903B have been found inserted into the *mgrB* gene of *K. pneumoniae* (Nirwan et al., 2021; Silva et al., 2021). These IS share high identity to their respective reference IS elements identified on plasmids. Furthermore, multiple IS insertion sites in *mgrB* (Zaman et al., 2018), coupled with the experimentally determined inducible colistin resistance arising from bacterial cells transformed with plasmids containing IS elements (Yang et al., 2020) and the presence of functional transposase genes detected among the IS elements in plasmids provide further evidence for the possible role of plasmids as donors of IS elements with the capability for *mgrB* insertion and gene inactivation.

The IS elements, ISKpn25, ISKpn26, ISKpn14 have a majority percentage prevalence within *K. pneumoniae* across the sampled data set, while *K. pneumoniae* represents the single largest species group carrying the IS903B element. The IncFIB(pQil) plasmid Inc group was associated with the IS element ISKpn25. This association has been previously observed in carbapenem and colistin resistant *K. pneumoniae* isolates derived from independent samples from both Greece, Italy, and Colombia (Cienfuegos‐Gallet et al., 2017; Giordano et al., 2018). The ISKpn25 containing IncFIB(pQil) plasmid appears highly successful, present in up to 26 countries from the sampled data, while seven countries harboring ≥5 IncFIB(pQil) ISKpn25 samples have been detected. These countries include India, the United States, Italy, Thailand, Germany, Canada, and Poland. From the countries with ≥5 samples, 94.2% (*n* = 64/69), IncFIB(pQil) ISKpn25 samples are derived from clinical sources, suggesting the IS element ISKpn25 associated with IncFIB(pQil) plasmids are well established in clinical settings from geographically distinct areas. This may present a serious clinical threat, as selective colistin pressure in the hospital environment has been linked to the genetic transposition of IS elements in *K. pneumoniae* (Berglund et al., 2018; Yan et al., 2021; Yang et al., 2020). In this scenario, there may be few barriers to prevent inducible colistin resistance among bacterial isolates encoding ISKpn25 on their plasmids.

In contrast, IS elements ISKpn14 and ISKpn26 are most often identified from clinical samples from China. These results agree with recent molecular analyses which reveal the presence of ISKpn14 as the principle IS element disrupting the *mgrB* gene of colistin resistant clinical *K. pneumoniae* isolates from six hospitals across China (Yan et al., 2021). ISKpn26 is associated with IncFII or IncFII and IncR fusion plasmids. Interestingly, IncFII and IncR fusion plasmids, and IncR plasmids are the most commonly identified plasmid Inc groups from ISKpn14 carrying plasmids, suggesting these plasmid Inc groups are receptive toward IS element uptake and maintenance. In addition, both ISKpn26 and ISKpn14 containing plasmids also carried a high percentage of carbapenem resistant genes, a feature also detected in ISKpn14/ISKpn26 *mgrB* disrupted *K. pneumoniae* isolates from China (Yan et al., 2021). This may represent a worrisome clinical threat, based on evidence that CPKP isolates are successful in clinical settings. *K. pneumoniae* isolates are more likely to have a genetically nearest neighbor from the same hospital if they harbor carbapenemase genes (David et al., 2019). The clonal success imposed by carbapenemase presence may help disseminate IS elements. The combination of carbapenemase genes in tandem with IS may produce *K. pneumoniae* isolates which are difficult to treat. Beyond, carbapenemase genes, predicted resistance was detected for 23 additional antibiotics (Figure A1b). Plasmids encoding IS903B exhibited the lowest predicted resistance for 13/23 antibiotics relative to other plasmids encoding IS elements, likely reflecting their source isolation. Multidrug resistant IS‐carrying plasmids may constitute a serious clinical threat. In this scenario, resistance to antibiotics may lead to colistin based therapy. Following colistin treatment the IS element may be mobilized, potentially leading to extensively or pan‐drug resistant isolates.

Furthermore, The IncFII typed pHN7A8 plasmid was present in 70.8% (*n* = 85/120) and 26.7% (*n* = 32/120) plasmids carrying ISKpn26 and ISKpn14, respectively. Conjugation assays reveal IncFII ‐pHN7A8‐like plasmids are highly transferable (Sennati et al., 2016). Prokka annotation reveals the pHN7A8 plasmid (GenBank accession no. JN232517) possesses conjugative transfer machinery. Both the reference plasmid, and a representative sample of 10 IncFII pHN7A8 Inc typed plasmids carrying either ISKpn26 or ISKpn14, encoded the Tra conjugative transfer operon. The abundance of IncFII plasmids encoding ISKpn26 and ISKpn14 may be linked to efficient conjugation of IncFII host plasmids and may represent a further independent mechanism promoting the emergence of colistin resistance.

In contrast to the other IS elements investigated, IS903B was sourced from environmental sources more than the other IS elements investigated and contains the largest number of unique Inc groups. This may represent the divergence sources IS903B‐containing plasmids are derived from. Concordant with these results, 37.5% (*n* = 3/8) of *K. pneumoniae* samples derived from food sources from India harbored IS903B insertions in the *mgrB* gene, confirming environmental sources harbor IS elements which disrupt *mgrB* (Ghafur et al., 2019). Furthermore, the IS903B IS element has been detected from a bovine veterinary sample inserted into the *mgrB* gene of a *K. pneumoniae* isolate conferring colistin resistance (Kieffer et al., 2014). It is noteworthy, that plasmids carrying IS903B have fewer carbapenemase genes relative to the other IS elements investigated; this may reflect a clinical scenario whereby the selective pressure imposed in hospital environments engenders a situation whereby plasmids preferentially acquire carbapenemase genes in response to treatment regimens.

Whole genome sequencing (WGS) is being increasingly employed in bacterial genomics to support pathogen surveillance. The results indicate plasmid Inc groups and the country of origin for particular *mgrB* disrupting IS elements. Moreover, the relationship between IS elements and the cocarriage of carbapenemase genes and respective isolation source is exposed. Importantly however, other IS elements can also disrupt the *mgrB* gene including IS10R, ISEcp1, and ISKpn28 (Jayol et al., 2015; Yang et al., 2020; Zaman et al., 2018). Furthermore, IS elements can disrupt other chromosomal colistin‐associated genes including *crrCAB*, giving rise to colistin resistant *K. pneumoniae* (Yang et al., 2020). Functional studies involving IS elements in plasmid vectors transformed into colistin susceptible *K. pneumoniae* and other Enterobacteriaceae isolates will also more fully reveal the role of IS‐carrying plasmids in the induction of colistin resistance. The discovery of the cocarriage of carbapenemase genes with IS elements in clinical samples indicates *K. pnuemoniae* strains are primed for resistance towards last‐resort antibiotics, limiting already narrow therapeutic treatment options.

## CONFLICT OF INTERESTS

None declared.

## ETHICS STATEMENT

None required.

## AUTHOR CONTRIBUTIONS

**Stephen Mark Edward Fordham:** Conceptualization (lead); data curation (lead); formal analysis (equal); investigation (lead); methodology (lead). **Anna Mantzouratou:** Project administration (equal); supervision (equal); validation (equal); writing–review & editing (equal). **Elizabeth Sheridan:** Investigation (equal); methodology (equal); project administration (equal); supervision (equal); writing–original draft (equal); writing–review & editing (equal).

## Data Availability

All data are provided in this article and its appendices, as well as in supplementary material available at https://doi.org/10.5281/zenodo.5812148

## APPENDIX A

### A.1

See Figure A1, Figure A2, Figure A3, and Table A1.

**Table A1:** IS903B, ISKpn14, ISKpn25, and ISKpn26 insertion sequence elements in the *mgrB* gene in *Klebsiella pneumoniae*

Insertion sequence element	Chromosomal *mgrB* insert (if specified)	Number of isolates	ST	References
IS903B	+70	1	ST37	Leung et al. (2017)
IS903B	−35, −21, −10, +15, +44, +53, +60, +70, +75, +88	10	N/S	Da Silva et al. (2021)
1S903B	+44	1	ST11	Yang et al. (2020)
				
ISKpn14	+127	1	ST101	Poirel et al. (2015)
ISKpn14	N/S	1	N/S	Halaby et al. (2016)
ISKpn14	−12 (2), +52 (4), +22 (2)	10	ST16 (1), ST14 (2),	Zaman et al. (2018)
			ST48 (1), ST15 (2), ST101 (3),	
			ST307 (1)	
ISKpn14	+119	1	ST11	Yang et al. (2020)
ISKpn14	N/S	7	ST11	Yan et al. (2021)
ISKpn25	+70	2	N/S	Haeili et al. (2017)
ISKpn25	N/S	1	ST258	Pitt et al. (2018)
ISKpn25	+133 (10)	10	ST258 (8),	Giordano et al. (2018)
			ST512 (2)	
ISKpn25	N/S	19	ST512	Cienfuegos‐Gallet et al. (2017)
ISKpn26	+75	2	ST258	Leung et al. (2017)
ISKpn26	+75	8	ST258	Pitt et al. (2018)
ISKpn26	+75	1	ST147	Pitt et al. (2018)
ISKpn26	+75	6	ST258 (2),	Giordano et al. (2018)
			ST512 (4)	
ISKpn26	+74 (8)	8	ST11	Yang et al. (2020)
ISKpn26	+75	1	ST395	Nirwan et al. (2021)
ISKpn26	+74	1	N/S	Da Silva et al. (2021)
